# Methods to Reduce Forest Residue Volume after Timber Harvesting and Produce Black Carbon

**DOI:** 10.1155/2017/2745764

**Published:** 2017-03-09

**Authors:** Deborah S. Page-Dumroese, Matt D. Busse, James G. Archuleta, Darren McAvoy, Eric Roussel

**Affiliations:** ^1^USDA FS, Rocky Mountain Research Station, 1221 S. Main, Moscow, ID 83843, USA; ^2^USDA FS, Pacific Southwest Research Station, 1731 Research Park Dr., Davis, CA 95618, USA; ^3^USDA FS, Umatilla National Forest, 72510 Coyote Rd, Pendleton, CA 97801, USA; ^4^Utah State University, 5230 Old Main Hill, Logan, UT 84332, USA; ^5^Nevada Division of Forestry, 2478 Fairview Drive, Carson City, NV 89701, USA

## Abstract

Forest restoration often includes thinning to reduce tree density and improve ecosystem processes and function while also reducing the risk of wildfire or insect and disease outbreaks. However, one drawback of these restoration treatments is that slash is often burned in piles that may damage the soil and require further restoration activities. Pile burning is currently used on many forest sites as the preferred method for residue disposal because piles can be burned at various times of the year and are usually more controlled than broadcast burns. In many cases, fire can be beneficial to site conditions and soil properties, but slash piles, with a large concentration of wood, needles, forest floor, and sometimes mineral soil, can cause long-term damage. We describe several alternative methods for reducing nonmerchantable forest residues that will help remove excess woody biomass, minimize detrimental soil impacts, and create charcoal for improving soil organic matter and carbon sequestration.

## 1. Introduction

Many forest stands in the western United States are in need of restoration for a variety of attributes (e.g., fire regimes or watershed health) after 100 years of fire suppression, selective harvesting, or livestock grazing [1–3]. Although there is broad agreement that some form of restoration of fire regimes, habitat, fish, and wildlife populations, or disturbance patterns is necessary in many areas of the western United States [4], there is disagreement about the objectives and implementation strategies [3]. In this paper we will consider slash disposal activities resulting from thinning operations that are used to reduce the volume of standing timber on a site. Stand density restoration activities usually involve cutting and removing small trees with little merchantable value [3]. Residues created from thinning activities designed to reduce wildfire were estimated to be approximately 0.2 million metric tons annually in the forests of Southern California and were expected to increase to 1,500 metric tons per day [5]. To reduce the risk of wildfire, residues are often removed and transported to a bioenergy facility, dispersed across the harvest site by masticating or grinding them, or piled and burned [6, 7].

Slash pile burning can be an economical method for disposing of harvest residues on National Forests following timber harvesting operations [8] and an effective method for reducing the volume of unmerchantable material. However, the impact of pile burning on soil processes is highly variable and can result in either relatively small impacts for a short period of time or long-term residual soil damage [9], but the ecological impacts are not well understood [2, 10, 11]. The high variability of soil impacts from pile burning impacts can be attributed to differences in soil texture, fuel type and loading, soil moisture, and weather conditions during burning (e.g., [12–14]). Often, slash piles leave only localized soil impacts; however depending on postharvest woody residue abundance, pile size, amount, and type of fuel in the piles, soil type, fire duration, and the distribution of piles within an activity area larger-scale impacts are possible [2, 15]. Alternatives to slash pile burning are limited and broadcast burning is often restricted by weather conditions, stand species composition, availability of expert fire crews, or air quality regulation that limit seasonal burning. Some areas are not suited for pile or broadcast burning and therefore, mastication (reducing the size of woody residues) is gaining popularity in many areas because it can be less expensive than burning. However, it does not remove fuels, it just rearranges them [16].

We briefly discuss the impacts of slash piles, how slash piles are currently built, and then discuss alternative methods for using waste woody residues to create biochar. Our paper is designed to provide information on the usefulness of making and applying biochar (or black carbon), purposefully made charcoal for land application. Purposeful biochar applications can be a vehicle for carbon sequestration made from renewable and sustainable woody biomass, but it can also help improve soil conditions by improving soil water and nutrient holding capacity [11].

## 2. Slash Pile Impacts

Determining the impacts of pile burning on soil health is complex because of the wide variability in how piles are constructed and distributed within a harvest area, amount of biomass to dispose, piling method, species composition, and pile location. In addition, soil is not a particularly good conductor of heat owing to its high internal porosity [17]. For example, hand-built pile coverage in a Lake Tahoe Basin study ranged from 2% to over 30% within thinning units [7]. In northeastern Oregon, estimates for whole tree yarding and bulldozer-built piles are one on 4 ha (10 acres) while processing trees within a harvest area may result in one bulldozer-built or hand-built pile in every 0.4 ha (1 ac; personal communication; Kristin Marshall, Assistant Fire Management Officer, Umatilla National Forest, Heppner, OR). Commonly, harvest units have less than 15% pile coverage (median of 8%) and the actual ground coverages are highly correlated with the level of basal area reduction [8].

Because slash is concentrated into piles, heat is concentrated into a small area where it can alter soil structure [12], infiltration [18], nutrient cycling [19], soil pH [20], and microbial populations [21]. Pile burning can also impact understory plants, seedbanks, and water holding properties [2, 22, 23]. Many studies suggest that pile burning occur when soils are moist to limit detrimental soil heating [11, 13], despite the potential for biological damage that can result from burning piles when the soil is moist [24–26].

When slash piles are built using a bulldozer they are often a mixture of dense fuels, mineral soil, and surface organic horizons [13, 27]. Once ignited, the piles often burn very hot for an extended period of time [27] and can produce long-term soil impacts. Pile size also plays a key role in soil impacts [14]. Season of burning and under-pile soil moisture and texture will alter the extent of impacts (Table 1). In northwestern Montana, for example, spring burning of grappler-built slash piles on fine-textured soil resulted in increases in soil organic matter, carbon, and nitrogen. Fall burning of grappler-built piles when soil moisture was low resulted in loss of more than half of the organic matter, carbon, and nitrogen. There are methods to restore burn scars (e.g., wood chip mulches or soil scarification) [7, 28], but these efforts also add to overall increased site preparation costs.

## 3. Current Pile Construction Techniques

Slash piles are currently used as the preferred method for residue disposal because they can be burned at various times of the year, offer a larger margin of safety, and are relatively effective at removing woody residues. Pile burning has been used for many years and is often the preferred method to reduce harvest-generated slash. Piles can be constructed in a variety of ways, by hand, bulldozer, excavator (grappler), or log loaders. In Table 2 we describe several strengths and weaknesses of slash pile burning.

### 3.1. Hand Piles

Typically these piles are a loose stack of wood built by placing one piece of wood onto the pile at a time. No care is taken to elevate the pile from the ground, but typically the pile rests on a few supporting branches that elevate the pile. There is also little effort to densify the pile during construction; leaving many air voids. In some cases hand piles do not create detrimental soil impacts as a result of heating or the act of building the pile [8, 29], but if soil moisture is low or the piles are extremely dry, they can impact the underlying soil. Soil temperature spikes exceeding 500°C beneath wood-dominated hand piles, with lethal temperatures above 100°C for 3 days have been recorded [8]. Hand-built piles constructed from smaller diameter thinning slash also surpassed lethal temperatures for 24 hours in the surface soil [8]. Charcoal production from hand-built piles can be considerable, yielding a 2-fold increase in soil C content compared to preburn levels, but short-term, concomitant declines in soil quality indices (water infiltration, fungal and bacterial populations, and nitrate levels) were also detected [30].

### 3.2. Bulldozer

These piles are often very dense. Piles are pushed together and, when the pile is large, the bulldozer will ride onto the pile to further compact it. This action increases the density of the pile and may also lead to changes in soil under and near the pile as the dozer can compact, displace, or rut the soil. Depending on the use of a brush rake or the skill of the operator, the resulting pile may also contain displaced forest floor material or topsoil that becomes packed into the pile base. Occasionally, displaced topsoil buries wood in the pile resulting in reduced air reaching the charred wood and creating some charcoal, similar to mound-style kilns [31].

### 3.3. Grappler or Log Loader

This equipment can also create a dense pile for burning. Typically these piles are “cleaner” than those built using a bulldozer because residues are picked up rather than pushed into a pile. In addition, the equipment operator has more control over the placement of woody residues. Instead of residues pushed into a pile, they are lifted and placed on the pile. However, similar to the dozer, excavators or grapplers can drive onto the pile or force the pile into a more compact form by using its boom and grapple resulting in more fuel in contact with the soil. However, the size of the material added to the pile is critical to how the pile will burn and the heat pulse into the soil [27]. Both dozer and excavator piles are often built on compacted landings which can increase the depth and intensity of the soil heat pulse during burning, in turn increasing detrimental impacts.

## 4. Making Biochar from Forest Residues

There has been increased interest in using woody residues generated from thinning or bioenergy harvests to make biochar. However, transportation costs to move unmerchantable woody material to a pyrolysis unit can be expensive, as can the pyrolysis equipment itself [32]. Therefore, creating biochar on-site can be less expensive and immediately applied back on a site as a soil amendment or to restore skid trails, log landings, or burned areas.

Traditional slash pile burning can result in some recalcitrant carbon (black carbon, biochar) produced under the burn area, but the amounts remaining depend on burn temperature, with black carbon originating at temperatures between 250 and 500°C [33]. Biochar is about 80% carbon [34] and less than 0.1% nitrogen [35], and its porous nature makes it potentially beneficial for increasing water holding capacity and decreasing bulk density [31]. It also alters cation exchange capacity and soil color and is the location of many ectomycorrhizal fungi [36]. Biochar can be used to restore soil function in areas where there is a loss of organic matter. One other potential use of forest residue-produced biochar is to augment lost soil organic matter in dryland farming [37, 38]. Charcoal forms naturally at a rate of 1–10% during wildfires [39]. On some sites, charcoal has been found dating back 11,000 years before present [40], but the quality of charcoal and its' recalcitrance is dependent on climate, soils, and plant species. Current efforts to convert biomass that would normally be burned in slash piles to biochar can result in 10–35% by volume inputs of carbon into the soil. This carbon is more stable and has a lower risk of releasing CO_2_ or other greenhouse gases into the atmosphere [41]. Amending sites with biochar during farming production or on forest sites after harvesting further protects biochar from degradation as it becomes part of the stable carbon pool [42].

In the next section, we outline methods that can be much less expensive than typical pyrolysis and deliver a high-carbon product that can be used to amend the soil.

## 5. Burning Slash Piles and Creating Biochar

**Figure 1:**
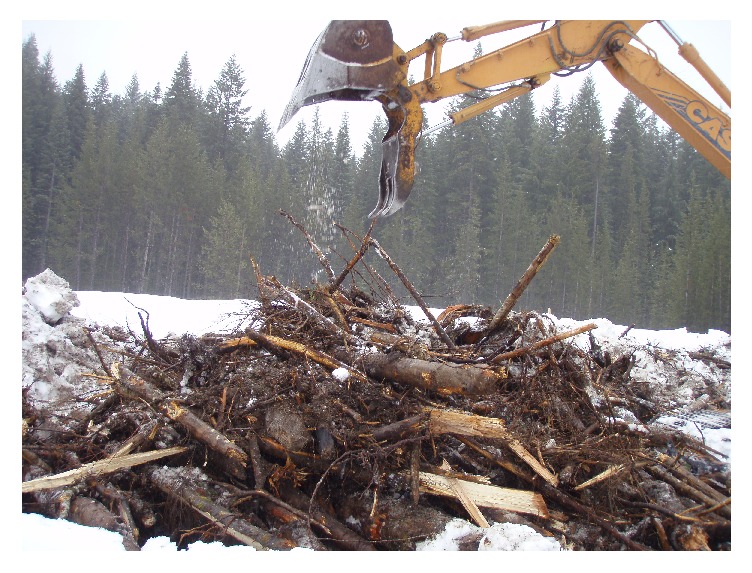
Elevated machine pile being constructed. (Photo credit: J. G. Archuleta.)

**Figure 2:**
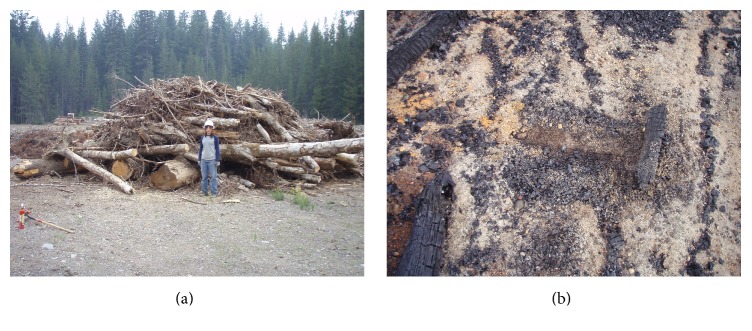
Finished burn pile (a) and biochar (b). (Photo credit: J. G. Archuleta.)

We developed an alternative method for building slash piles to reduce the amount, extent, and duration of soil impacts from burning and create more charcoal for use in soil restoration in or near the piles. To maximize the creation of charcoal the burn pile was elevated above the soil surface on large logs, with smaller material piled perpendicularly on top (Figure 1). Grapplers were then used to build a pile on the base logs. There are several advantages to elevated piles: (1) potential for greater air flow to dry woody material, (2) limited moisture wicking up from the soil into the wood, (3) construction time is similar to other only pile-building methods, and (4) potential to limited soil impacts to the areas where the base logs are in contact with the soil (Figure 2). Base logs for this type of slash pile can be as small as 10 cm (~4 in) in diameter and still provide protection to the soil.

We estimate that approximately 10–15% of the volume of wood in the pile can be converted to charcoal but is dependent on environmental and pile properties when burning. Production of biochar from this type of pile can be raked into the soil around the burn area for restoration of compacted soils or to provide additional organic matter near the pile. See Table 3 for information on carbon and nitrogen produced in slash piles.

## 6. Other Methods to Create Biochar

Kilns have been used for centuries to make charcoal. Often built as earth-covered pits or mounds, traditional kilns provided an inexpensive, efficient means for charcoal making [43]. Other kilns have been made of brick, metal, or concrete [33]. Kilns operate in batch mode in which feedstock is added and charcoal is removed. However, newer kilns can provide automatic feed (see the rotary kiln description below).

### 6.1. Metal Kiln

**Figure 3:**
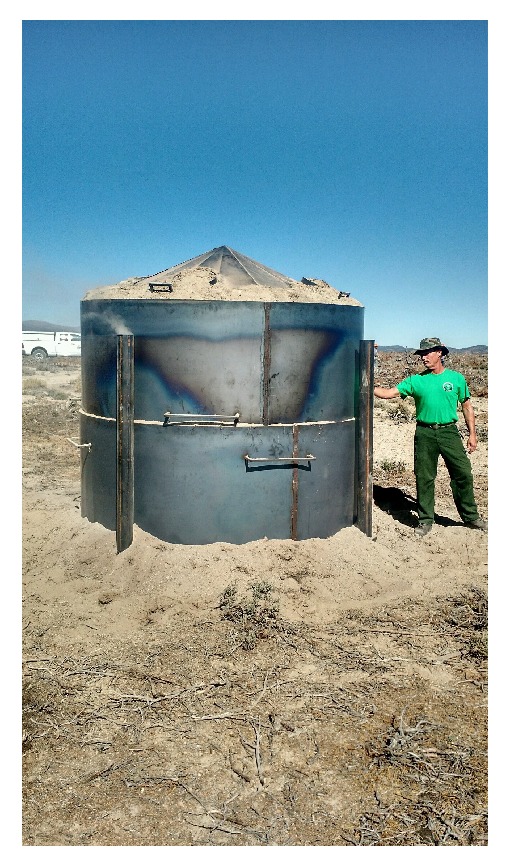
Metal kiln being used to process piñon and juniper woody biomass. (Photo credit: E. Roussel.)

Kilns made of metal were designed to be relatively portable [44]. They have two cylindrical sections and a conical cover with four steam release ports and the bottom section sits on four inlet ports. Air flow into and smoke out of kiln can be controlled through the ports so that both charcoal quantity and quality can be controlled. The kiln shown in Figure 3 can hold approximately 8 cubic meters (10 cubic yards). During production, wood biomass is reduced by approximately 65%. One batch takes approximately 2 days to complete which includes loading the kiln, lighting the fire, adding the chimneys, and closing off the inlet ports. Multiple kilns at one site can process the residues more efficiently. Because the kiln is constructed in section, it can be loaded onto a trailer for transport to the harvest site. Metal kilns can be used in remote areas accessible by a pickup truck and the feedstock needs little postharvest processing, such as chipping. In addition, unskilled personnel can be quickly trained to operate the kiln. Charcoal produced from this kiln has approximately the same dimensions as the wood that was put into it. However, the charcoal fragments easily and driving over it with a large truck shatters the charcoal to make it easier to spread. See Table 3 for an example carbon and nitrogen data from this type of biochar production.

### 6.2. Rotary Kiln

**Figure 4:**
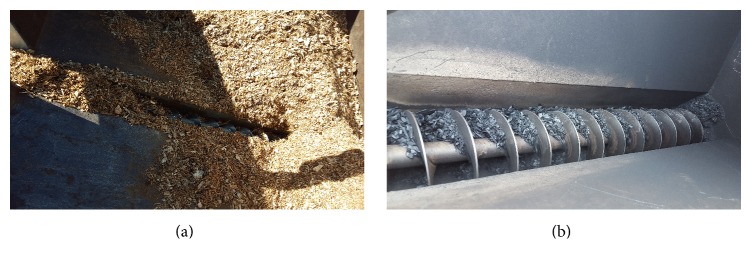
Rotating auger moving (a) chips and (b) biochar in the rotary kiln. (Photo credit: S. Bell, retired USDA Forest Service.)

Rotary kilns were developed for large-scale forest harvest operations which generate large volumes of woody residue [45]. A rotating metal tube is heated from the outside with gas burners to temperatures of 400 to 600°C (Figure 4). The tube is in constant motion which quickly exposes woody residues to extreme temperatures, allowing the feedstock (wood chips) to be rapidly heated. The extreme heating of small particles in a low oxygen environment quickly transforms the wood into three potentially high-value products biochar, biooil, and syngas. At times, biochar is the targeted output, but for other applications biooil may be the desired output.

The entire rotary kiln unit is housed in a shipping container or trailer making it relatively portable into a forest environment. It also requires a trailer to move supporting equipment that includes hoppers and feed bins, a high-lift forklift, and an electrical generator.

Rotary kilns can process up to 18 Mg (20 tons) of feedstock in 24 hrs. The ideal chip size is 1.3 cm (1/2 in) or less, to maximize throughput. It is also ideal to have the feedstock as dry as possible, less than 10 percent moisture. The machine will function when the feedstock is very wet and wood particle size is up to 5 cm (2 in), with the throughput and char quality significantly reduced and an increased risk that a large wood piece will damage the equipment. The dimensions of the feedstock remain unchanged through the pyrolysis process biochar looks similar to the chips except they turn black after processing. When focused on biochar production for agriculture it is most desirable to have small, consistently sized feedstock so the material will mix well with soil or be deployed using a lime spreader or other agricultural spreader-type equipment. In forest operations, the biochar does not have to be uniform and can easily be spread on slopes, log landings, or skid trails using the biochar spreader [46].

In addition to being relatively mobile, another advantage of the rotary kiln is the control it offers the operator. Adjusting the temperature and the time the wood chips are in the kiln will produce biochars of different qualities. Biochar can be more effective if its chemistry is designed to target specific soil quality issues [47]. For example, in locations where crop yield increases are not a goal, biochar can be used to sequester carbon. However, improving water holding capacity, infiltration, or nutrient retention may be achieved by biochar designed for these purposes [48]. Biochar made in kilns tend to have higher carbon and nitrogen contents than biochar from slash piles or the air curtain burner (Table 3).

### 6.3. Mini Kiln

**Figure 5:**
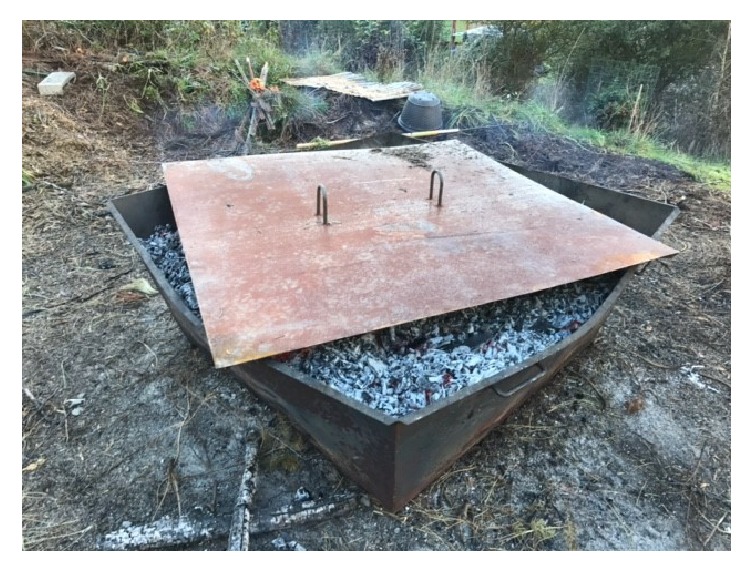
Mini kiln with charcoal ready to be covered to create biochar. (Photo credit: D. Morrison, retired USDA Forest Service.)

These simple, low cost kilns are operated primarily by family forest owners (generally < 500 acres) interested in conservation stewardship of their land. The appeal comes from recognizing the benefits of biochar as a soil amendment and as a mechanism to sequester carbon from the atmosphere, along with a desire to seek alternative means of managing thinning residues besides pile burning. A main attribute of the mini kiln is its light-weight construction for easy transport by 1-2 people. Design characteristics of the kiln (shape, volume, and thickness of metal walls) are user defined, often by a trial-and-error process. An example of mini kiln construction is provided by the Umpqua Biochar Education Team (http://ubetbiochar.blogspot.com), which is essentially a truncated and inverted pyramid with an open top and a narrower base that rests on the forest floor. A drain plug is installed near the base to release any water from the quenching process. Thinning residues are cured for a year or more, placed in the open kiln, and burned, and then the coals are either quenched with water or by covering with a metal lid to deplete the oxygen source (Figure 5).

The advantages of mini kilns are their low cost, ease of use, and transportability. Because of the relatively small scale of this operation, the quantity of biochar produced is generally limited, and the products are often used for improving soil tilth of nearby gardens, small orchards, or pastures (Personal communication, Don Morrison; retired Forester with the USDA Forest Service). Again, this operation is geared to meet the needs of small-land owners; efforts to scale-up the use of mini kilns to treat thinning residues on a stand-level basis are of growing interest and will likely hinge on the economic feasible of biochar production relative to pile burning.

### 6.4. Air Curtain Burner

**Figure 6:**
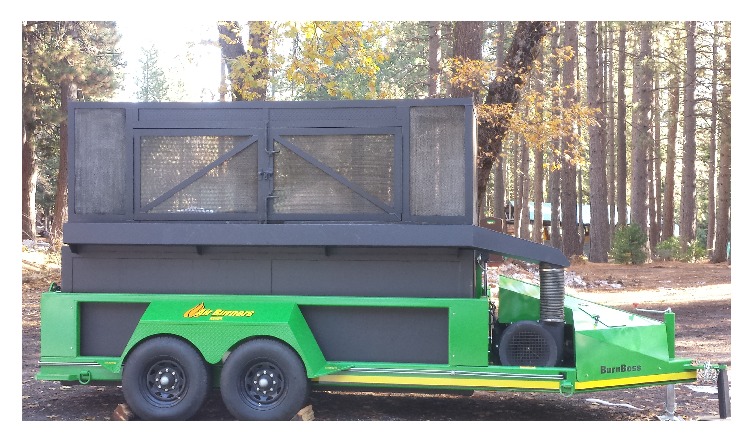
Air curtain burner (Photo credit W. Jang, Humboldt State University.)

These burners are designed to dispose of woody residues as an alternative to open burning (slash piles) and were developed to be used near large-scale harvest operations generating large volumes of woody residues (Figure 6).

The mechanics and operation of the air curtain burner are described by at https://airburners.com/DATA-FILES_Tech/USDA-FS-Tech_Tips-0251-1317pr.pdf. In general, air curtain burners can quickly dispose of freshly cut as well as dried material; disposal rates are typically 1 to 9 Mg (1 to 10 tons) per hour depending on the size and capability of the equipment. Similar to the kilns, large trees and brush can be loaded into the burner in batches without the need for chipping. In addition, the burner has few moving parts and reaches a high temperature. Since the air curtain burners usually burn very hot, the residue remaining is ash rather than biochar. See Table 3 for an example of the carbon and nitrogen content of charcoal created with this method.

The current trend towards using woody biomass for the creation of biochar comes from the Amazonian Terra Preta soils which have a higher soil fertility believed to be the result of intentional black carbon additions from slash and char agriculture [49]. Of the techniques listed above for creating biochar, building slash piles to move the heat pulse away from the soil is the easiest since grapplers or other equipment are already on-site. However, in many areas this method is not feasible or practical. For example, a small wood-lot owner would be more likely use the mini kiln method for a low volume of wood and the charcoal moved with small farm equipment. Both the metal and rotary kilns are useful on many sites, but they are currently used to remove invasive species or dense stands of piñon-juniper. The air curtain burner has been most effective where there are large amounts of woody residue and where the use of open slash pile burning is limited. Soils low in organic matter (e.g., coarse-textured, degraded agricultural land, burn piles, and skid trails) are all areas that would benefit from the application of biochar [11]. In addition, it is important to know what biochar properties are important for individual soil restoration activities. Biochar carbon and nitrogen (Table 3) concentrations are important, but other properties such as pH, particle size, or electrical conductivity may also be critical attributes.

## 7. Forest Management Implications

Currently, forest restoration or rehabilitation treatments involve forest thinning and regeneration harvests that can produce 40–60 million dry metric tons of woody biomass per year [50]. Forest thinning operations, coupled with creating and spreading biochar, benefit both soil and forest health. Unlike agricultural soils where biochar can be added and tilled into the soil profile, application of biochar on forest sites is more difficult since trees, stumps, and downed wood hinder equipment movement across a site. However, in managed forests log landings, skid trails, abandoned roads, or abandoned mine land soils all require some form of restoration. Using a biochar spreader [27, 46] on these types of soil and sites is an ideal way to spread locally created biochar.

Given the large volumes of woody biomass created during harvesting in many forests, excess biomass may be converted to biochar and used by agricultural producers. This biochar creates a new market for timber purchasers to consider when bidding on harvest units. In addition, with the more wide-spread use of kilns and other methods to create biochar, areas with dead or unmerchantable timber from drought, disease, insect, or wildfire may be a feedstock source for biochar production and help lessen the future risk of wildfire.

Many North American forests face management challenges related to wildfire, insect and disease outbreaks, and invasive species resulting from overstocked or stressed stands. These forest stresses are already being exacerbated by climate change [11, 51] and therefore, creating and amending soil with biochar may be one method to mitigate soil drought conditions and sequester carbon [11].

## Figures and Tables

**Table 1:** Mean slash pile size, soil moisture, and resulting changes in soil properties to a depth of 30 cm after slash piling burning in two seasons on two soil textures relative to the control, unburned soil at the Lubrecht Experimental Forest, Montana.

Soil texture	Burn season	Soil moisture %	Pile size Mg	pH	OM	C	N
				% change from unburned			
Coarse	Spring	16.7	7.2	+9	−49	−50	−56
	Fall	11.8	9.8	+25	−64	−57	−63
Fine	Spring	30.0	9.6	+9	+10	+18	+17
	Fall	12.6	5.6	+12	−39	−25	−3

**Table 2:** Strengths and weaknesses of slash pile burning.

Strengths	Weaknesses
Widely used for many years	Soil heating damage; changes in chemical, physical, and/or biological properties
Easily controlled fire	Smoke, greenhouse gases, and particulates released
Relatively inexpensive form of site preparation or fuel reduction	Visual scars
Longer available time frame for burning	Invasive species increase

**Table 3:** Carbon and nitrogen content of biochar created using pyrolysis and some low-technology methods.

Feedstock	Product	Process	Carbon	Nitrogen
			Percent	
Mixed conifer	Biochar	Fast pyrolysis	86	0.18
Piñon-juniper	Biochar	Metal kiln	76	0.50
Mixed conifer	Ash and char mixed	Slash pile	28	0.22
Mixed conifer	Ash	Air curtain burner	48	0.37
Russian olive	Biochar	Rotary kiln	73	1.69
